# A Low-Cost Palmtop High-Speed Capillary Electrophoresis Bioanalyzer with Laser Induced Fluorescence Detection

**DOI:** 10.1038/s41598-018-20058-0

**Published:** 2018-01-29

**Authors:** Jian-Zhang Pan, Pan Fang, Xiao-Xia Fang, Ting-Ting Hu, Jin Fang, Qun Fang

**Affiliations:** 1 Institute of Microanalytical Systems, Department of Chemistry and Innovation Center for Cell Signaling Network, Zhejiang University, Hangzhou, 310058 China; 2 Department of Cell Biology, Key Laboratory of Cell Biology, Ministry of Public Health, and Key Laboratory of Medical Cell Biology, Ministry of Education, China Medical University, Shenyang, 110001 China

## Abstract

In this work, we developed a miniaturized palmtop high-speed capillary electrophoresis (CE) system integrating whole modules, including picoliter-scale sample injection, short capillary-based fast CE, high-voltage power supply, orthogonal laser induced fluorescence (LIF) detection, battery, system control, on-line data acquisition, processing, storage, and display modules. A strategy of minimalist miniaturization combining minimal system design and low-cost system construction was adopted to achieve the instrument miniaturization with extremely low cost, which is differing from the current microfabrication strategy used in most reported miniaturized CE systems. With such a strategy, the total size of the bioanalyzer was minimized to 90 × 75 × 77 mm (length × width × height) and the instrument cost was reduced to ca. $500, which demonstrated the smallest and lowest-cost CE instrument with LIF detection in so far reported systems. The present bioanalyzer also exhibited comparable analytical performances to previously-reported high-speed CE systems. A limit of detection of 1.02 nM sodium fluorescein was obtained. Fast separations were achieved for multiple types of samples as amino acids, amino acid enantiomers, DNA fragments, and proteins with high efficiency. We applied this instrument in colorectal cancer diagnosis for detecting KRAS mutation status by polymerase chain reaction-restriction fragment length polymorphism (PCR-RFLP) method.

## Introduction

Currently, miniaturization of analytical instruments with the aim of application in field analysis, point of care testing, environmental analysis and aerospace analysis, has become one of the main trends of analytical instrument researches. Since 1990s, the appearance of microfluidic technology has provided a strong impetus for analytical instrument miniaturization due to its advantages of high efficiency, high throughput, low consumption, as well as system miniaturization, integration, and automatization. By now, it has already become the major approach for achieving miniaturization of analytical instruments. Various microfluidic chip-based miniaturized analytical systems have been developed, including high-speed capillary electrophoresis (CE)^1–3^, microchip-based nucleic acid analysis^4,5^, centrifugal microfluidic immunoassay^6^ and high-performance liquid chromatography^7^.

High-speed CE which was first reported by Jorgenson’s group in 1991^8^, is a type of CE technique with features of high separation speed and high separation efficiency over traditional CE technique^9^. A typical high-speed CE system usually can achieve fast sample separation within tens of seconds using short separation length (<15 cm), narrow injected sample plug (e.g. <100 μm), and high separation electric field strength (>500 V/cm), while keeping high separation efficiency up to micrometer or submicrometer plate heights. Various high-speed CE systems have been developed and applied in biological^10,11^, medical^12,13^, chemical^14,15^, and environmental^16,17^ analysis. Currently, the miniaturization of high-speed CE systems has become one of the major development directions of the high-speed CE technology, which can provide various portable instruments^18,19^ for point of care testing^20^, *in-situ* analysis^21^ and extraterrestrial exploration^22^ by means of their small size, high-resolution separation and fast analysis time. So far, most of miniaturized high-speed CE systems^2,20,23–27^ are developed on the basis of microchip-based CE technique with advantages of automated picoliter-scale sample injection and separation and high system integration. In the early stage of the development of miniaturized CE instruments, electrochemical detectors were adopted frequently due to their simple structure and small size. In 2003, Jackson *et al*.^23^ developed a miniaturized CE instrument based on amperometric detection. It integrated a glass CE chip, battery, dual-source high-voltage power supply, interface circuit and modules, with a total size of ca. 100 × 150 × 25 mm. Becker *et al*.^24^ developed a portable CE instrument (190 × 120 × 80 mm dimensions) with a disposable polymethyl methacrylate chip and capacitively coupled contactless conductivity detector for on-site measurement of Li^+^, Na^+^, K^+^ in food analysis. Floris *et al*.^20^ reported a CE chip-based device for point-of-care testing, which consisted of a hand-held analyzer with conductometric detection (120 × 60 × 25 mm dimensions) and a small disposable cartridge containing the CE chip. The device was used to measure the lithium concentration in blood for the monitoring and treatment of patients with bipolar disorder. Fernandez-la-Villa *et al*.^25^ developed a battery-powered portable electrophoresis instrument (150 × 165 × 95 mm dimensions) for *in-situ* applications using single- and dual-channel SU-8/Pyrex microchips with electrochemical detection. The chip-based CE separation of dopamine and 3,4-dihydroxy-L-phenyl-alanine was completed with time of 32 s and separating efficiency of ca. 16000 and 37000 N/m, respectively. Compared with electrochemical detection, laser-induced fluorescence (LIF) detection technique has high detection sensitivity, high spatial resolution, and fast response time. Therefore, although it still presents great challenges in achieving miniaturization of LIF detectors due to their complex optical structure, currently LIF detection has become the major detection technique used in miniaturized CE instruments. Renzi *et al*.^2^ reported a handheld LIF-based analytical instrument, named μChemLab, for detection and identification of proteins and other biomolecules. The instrument integrated a microchip and a miniaturized LIF detection module, as well as high-voltage power supplies, electronic controls, data algorithms, and a user interface into a case of 115 × 115 × 190 mm. The LIF detection module had picomolar (10 pM) detection limit for fluorescent dyes and nanomolar (1 nM) for fluorescamine-labeled proteins. Meagher *et al*.^26^ also developed a portable LIF-based diagnostic device for detecting biological toxins (0.1‒10 nM detection limit) in bodily fluids based on the on-chip immunoelectrophoretic separation. The device integrated glass microchip, high-voltage power supply, confocal LIF detector with 532/633 nm laser and photomultiplier (PMT), and electronic control, processing and display modules with a total size of approximately 230 × 200 × 130 mm. Despite the great success of the above-mentioned miniaturized microchip-based high-speed CE analyzers in system integration and miniaturization, the relatively high cost and complexity in system structure, building, and controlling due to the use of microfabricated chips may limit the broad application of these systems in routine laboratories.

Actually, high-speed CE analysis can also be carried out alternatively on the basis of a short capillary by using special picoliter-scale injection methods such as optical gated injection^28^, flow gated injection^29,30^, and translational spontaneous injection^31,32^ methods to maintain high separation efficiency. Recently, we developed a compact high-speed CE bioanalyzer integrated modules of sample injection and changing, short capillary-based CE separation, LIF detection, as well as a custom designed tablet computer for data processing, instrument controlling, and result displaying with a desktop instrument size of 230 × 170 × 190 mm^33^. Compared with microchip-based high-speed CE systems, the use of cheap and commercially available fused silica capillaries can reduce system cost and simplify system structure. However, the miniaturization of this type of high-speed CE systems is still presented major challenge in miniaturizing all of the modules in the system, especially the picoliter-scale injection and detection modules.

**Figure 1:**
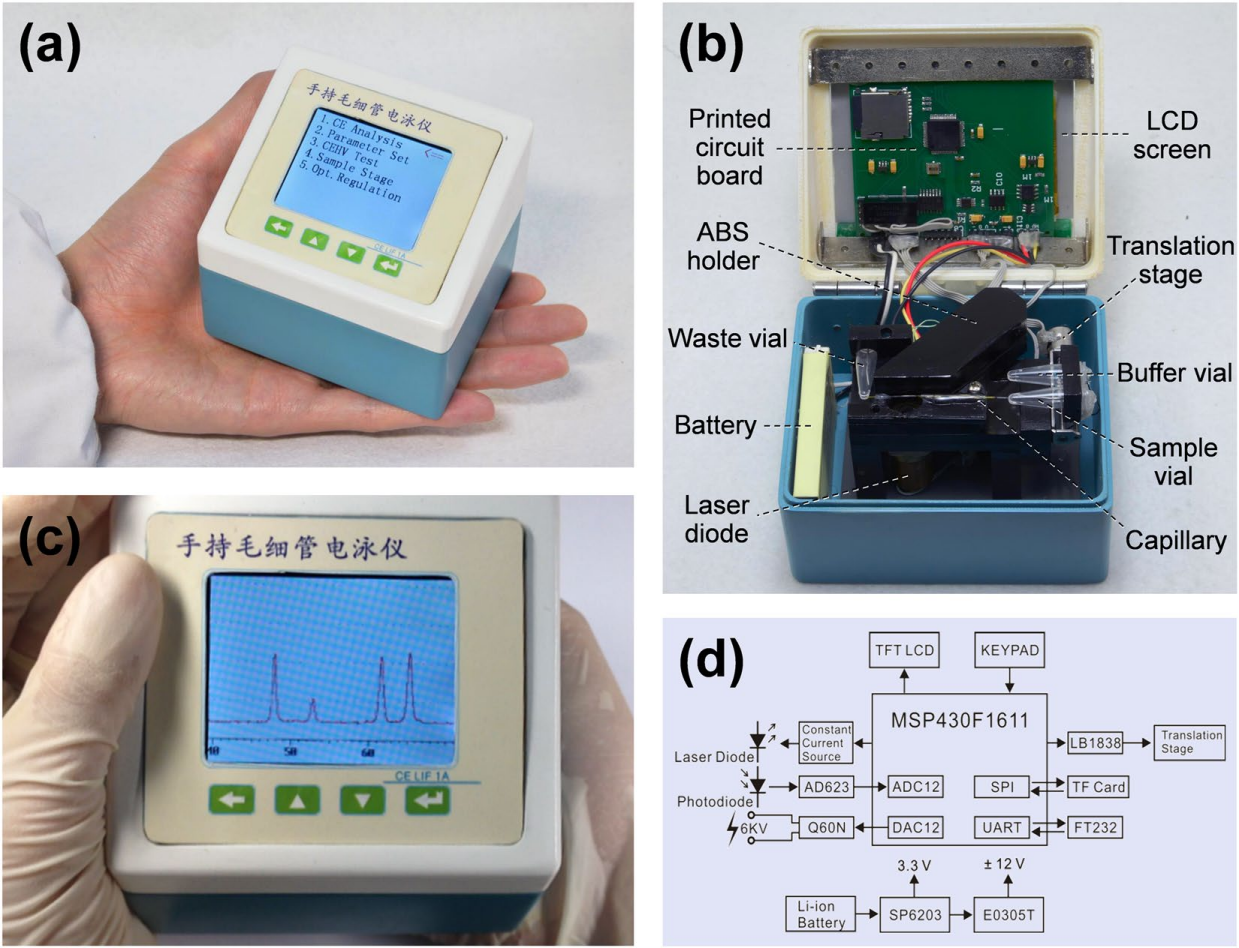
Images of the fully-integrated palmtop CE bioanalyzer. (**a**) Overall appearance; (**b**) Uncovered appearance; (**c**) An electropherogram of separation of amino acids (FITC-labeled arginine, phenylalanine and glycine) is displayed on the screen in real time; (**d**) Schematic diagram of total electronic module of the bioanalyzer.

**Figure 2:**
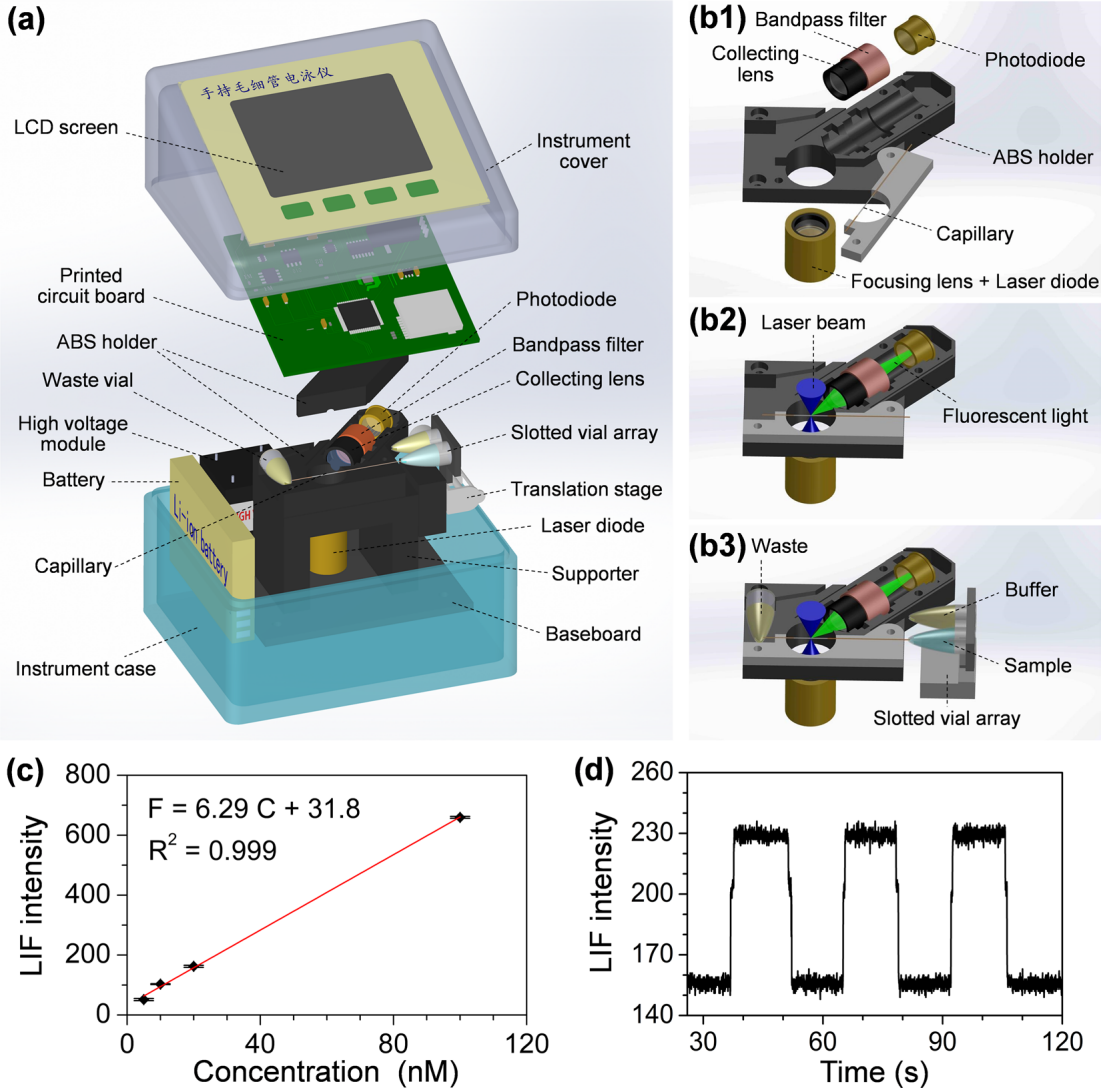
(**a**) Schematic diagram of the instrument structure; (**b1–b3**) Schematic diagram of the LIF module: (**b1**) Diagram of dispersed optical components, capillary and the special designed holder; (**b2**) Diagram of the assembled LIF module with a capillary; (**b3**) Diagram of the assembled LIF module integrated with a capillary and slotted vial array for CE injection and separation; (**c**) Linear relationship of fluorescence intensity to concentration in the test of limit of detection for sodium fluorescein. Sodium fluorescein concentrations: 5, 10, 20, 100 nM in 5 mM borate buffer (pH 9.2). (**d**) Typical recording of LIF intensity signals of 5 nM sodium fluorescein in the test of limit of detection.

In this work, we proposed a minimalist miniaturization strategy to further improve the miniaturization of a high-speed CE bioanalyzer to palmtop level and reduce the instrument cost by combining minimal system design and low-cost instrument components and construction. With this strategy, the total size of a high-speed CE bioanalyzer could be minimized to 90 × 75 × 77 mm (Fig. 1) and the instrument cost could be reduced to ca. $500, which integrated multiple modules including picoliter-scale translational spontaneous sample injection, short capillary CE, high-voltage power supply, orthogonal LIF detection (Fig. 2), battery, system control, on-line data acquisition, processing, storage, and display modules (Fig. 1). The performance of the instrument was demonstrated in multi-mode separations for amino acids, DNA fragments, amino acid enantiomers and proteins with fast speed and high efficiency, as well as in analysis of PCR products and restriction fragment length polymorphism (RFLP) digestion products of KRAS proto-oncogene for actual colorectal cancer diagnosis.

## Results

### Miniaturization Design of High-Speed CE bioanalyzer

Since the concept of micro total analysis systems (MicroTAS) was proposed by Manz *et al*. in 1990, microfluidic chip technique has become the major technique for achieving miniaturization of analytical systems. Despite the great success of microfluidic technique in developing miniaturized analytical systems, the microchip-based miniaturization strategy also faces some challenges. One of the challenges comes from the requirement of expensive equipment and complicated procedure in the microfabrication of many microchips, usually including the design and fabrication of microchannel network, chip bonding and chip surface treatment processes. In addition, to achieve the miniaturization of a total analytical system means all of the modules in the system should be miniaturized and integrated, including not only microchip but also other additional modules for ensuring the normal working of the whole system. For example, a CE analyzer should include high-voltage supply, detection, system control, data processing and result display modules. Currently, addressing these challenges mainly relies on the usage of costly, time-consuming and complicated microfabricated techniques.

In this work, we tried to use a different strategy to achieve the miniaturization of a high-speed CE system, namely minimalist miniaturization, which more relies on the use of minimalistic system design as well as low-cost elements and construction, rather than costly and complicated microfabrication techniques and devices, to build the microanalytical system, under the premise of ensuring comparable analytical performance. This would reduce the difficulty and expense in instrument miniaturization research and production, and thus may promote the widespread development and popularization of miniaturized analytical instruments.

For a high-speed CE system, the system miniaturization mainly lies in the miniaturization of three major components, sample injection, detection and overall electronic modules. Besides small size, the sample injection module should be capable of performing automated picoliter-scale sample injection with good repeatability. As for the detection module, simple structure, sensitive response and high resolution are the essential requirements. The overall electronic module is seldom mentioned in many articles in instrument development, while plays an indispensable role in achieving the miniaturization of the total analysis system. It should control the working states of all of the electronic modules in the instrument and avoid the possible interference between each other.

### Mini Picoliter-Scale Spontaneous Injection Module

In a high-speed CE system, for ensuring high speed and high efficiency separation, picoliter scale sample injection volumes are essential. So far, two types of picoliter-scale sample injection techniques based on short capillaries^28–32^ and microfluidic chips^34–36^ have been developed. The former ones include optical-gating injection^28^, flow-gating injection^29,30^, and translational spontaneous injection^31–33^ methods. The microchip-based injection methods include pinched injection^34,35^ and gated injection^35,36^ in cross channel or double-T channel. In most of the hitherto reported miniaturized high-speed CE systems, microchips were adopted because they could provide the microchannel networks for achieving picoliter-scale sample injection and CE separation, although this may lead to the evident increase in system cost and operation complexity. Differing from the nanoliter-scale injection methods commonly used in conventional CE systems, the capillary-based picoliter-scale injection techniques^28–33^ also provide an alternative strategy to achieve miniaturization of HSCE systems without the need of microfabricated chips. However, this strategy also brings difficulties in achieving system miniaturization since these systems need to use large-sized components, such as high-intensity laser for optical-gating injection^28^, flow switching device for flow-gating injection^29,30^, or mechanical translation stage for spontaneous injection^31–33^.

**Figure 3:**
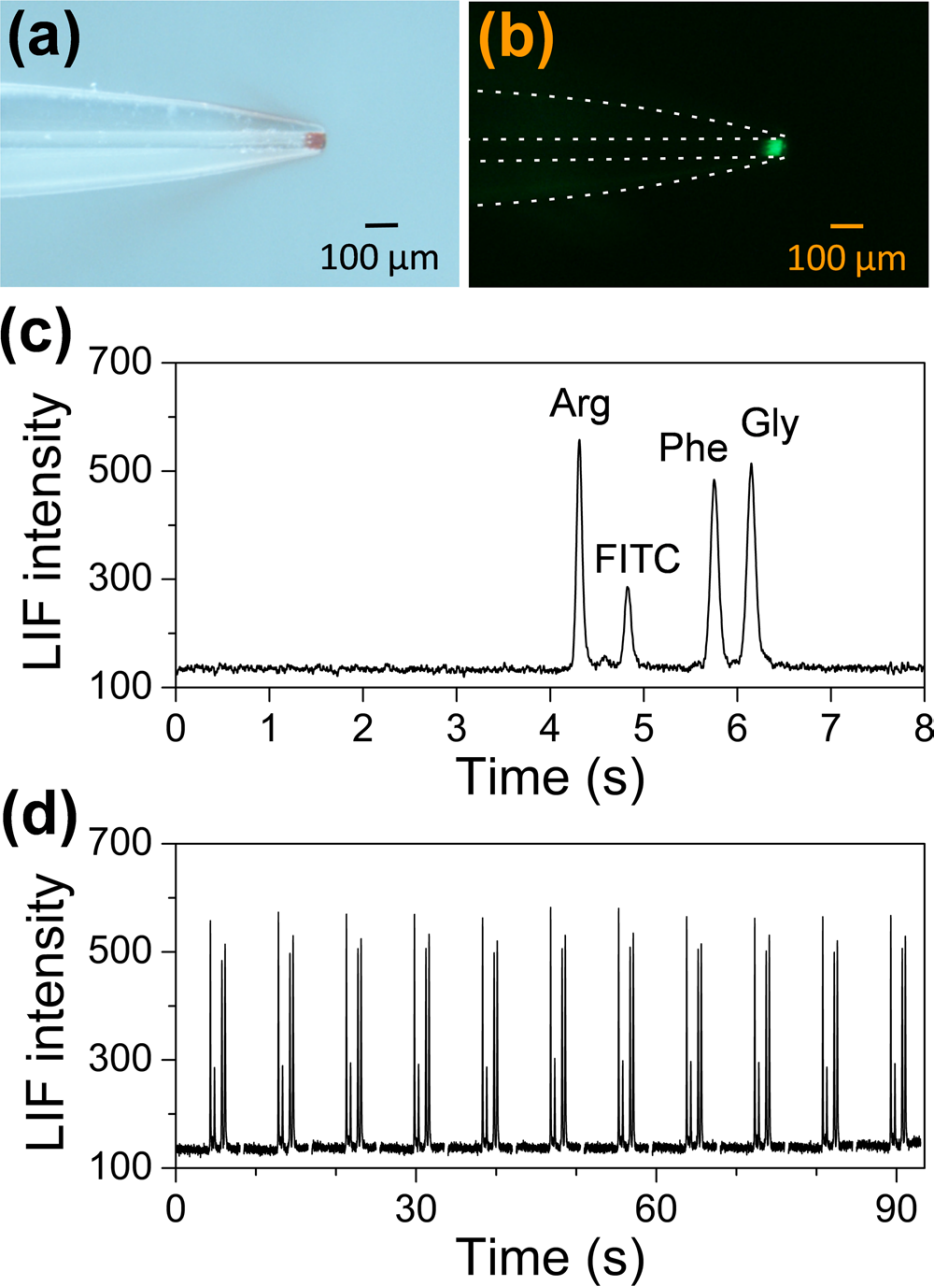
(**a**) Typical bright-field image of an injected sample plug (Red dye) at the tapered tip of a 50 μm i.d. capillary after translational spontaneous injection; (**b**) Typical fluorescence image of an injected sample plug of sodium fluorescein at the tapered tip of a 50 μm i.d. capillary after translational spontaneous injection; (**c**) Typical electropherogram of FITC-labeled amino acids; Conditions: sample, mixture of 5 μM FITC-labeled arginine, phenylalanine and glycine; working electrolyte, 5 mM borate buffer solution (pH 9.2); fused-silica capillary, 50 μm i.d., 3.8 cm total length, 2.6 cm effective separation length; translational spontaneous injection mode; separation field strength, 800 V/cm. (**d**) Electropherogram of 11 continuous separations of three FITC-labeled amino acids. Conditions are the same as (**c**).

In the previous study in high-speed CE, the authors’ group has developed a translational spontaneous injection method for performing picoliter-scale sample injection in a short capillary with a switchable slotted-vial array^31–33,37,38^. The translational spontaneous sample injection was performed by first inserting the tapered inlet end of a separation capillary into a sample reservoir, and then allowing the sample reservoir to remove from the capillary at an angle of 90° between the reservoir moving direction and the capillary. During this removing process, a small sample droplet in the picoliter range was remained at the tip end of the capillary, and was sucked into the capillary channel immediately by the surface tension of the sample droplet, achieving picoliter-scale sample injection. Compared to the other high-speed CE systems, our systems have relatively simple structure and are easy to build and operate, which provides favorable foundation for achieving system miniaturization. However, the large-sized translation stage elements (at least tens of centimeters) are required to be used in these systems for achieving automated switching of sample and buffer vials, which forms the major difficulty in achieving further miniaturization of the sample injection module. Actually, most of commercial translation stages used in various routine instruments were designed for high precision (~10 μm) and heavy load capability (~kg), and thus with large size and weight. However, such performances far exceed the requirement of the translational spontaneous injection method with tens micrometers and tens gram load. Thus, we chose a mini translation stage with a micro stepping motor instead of the large ones to switch sample and buffer vials, which was originally used in an autofocusing module of a digital camera with a minimal size of 36 mm (length) × 8 mm (diameter) as well as extremely low cost of $2. By coupling with the system control module, the movement of the mini stage could be automatically controlled with forward and backward direction in 17-mm effective moving distance with a positioning precision of ca.10 μm, which is sufficient for switching the sample and buffer vials. After optimizing the mini-stage moving velocity, step length, and interval time, the mini injection module could achieve picoliter-scale sample injection (e.g. 90 pL as shown in Fig. 3a and b) under the translational spontaneous injection mode, which substantially ensures the high speed and high efficiency performance of the present bioanalyzer. This module with low cost and significant size reduction of 2–3 orders of magnitude also demonstrated good working stability in continuous analysis (See section of “*Performance of the high-speed CE bioanalyzer*”).

### Orthogonal LIF Detection Module

Currently, two major types of optical arrangements for LIF detection, confocal and orthogonal arrangements, are widely used in CE systems. Confocal LIF systems usually have high sensitivity and high spatial resolution, however, their complexity and critical structure as well as difficulties in system construction appear to limit the miniaturization of the systems. In an orthogonal LIF system, the laser exciting and fluorescence collection paths are separated, which could effectively reduce the background signal without complex optical components. Such a system has simpler structure and is easier to be miniaturized.

For building the compact LIF detection module, we adopted three measures. First, we used optical components with size as small as possible, including a laser diode (18 mm length, 12 mm diameter) as light source, two small collimating lenses for laser beam focusing and fluorescence collection, and a photodiode (9 mm length × 6.2 mm diameter) as optical detector instead of a photomultiplier. The collimating lens consists of three lenses and has a small size of 8.7 mm length × 9.6 mm diameter and 7.0-mm focal distance. It has excellent focusing property, with which a focusing spot of 10 μm for the laser beam could be obtained at the center of the capillary channel. The photodiode was chosen due to its small size compared with normal or even miniaturized photomultiplier modules, though, which in turn would lead to the reduce in detection sensitivity of the LIF module. Second, to compensate the loss in sensitivity by using the photodiode and further improve the detection sensitivity, we employed a high-intensity laser diode (80 mW) to increase light source intensity and the 45° deviated orthogonal LIF detection mode^37^ to decrease the background scattering light intensity. In conventional orthogonal LIF systems, the fluorescence collection angle of 90° to the capillary at the plane perpendicular to the laser beam is commonly used. In our previous study^39^, we observed that such an angle was not an optimal solution. When changing the collection angle to 45° to the capillary at the same detection plane, the fluorescence signal to background noise ratio could be significantly increased due to the reduce of background signal and reach to a maximum value higher one order of magnitude than 90°. As a result, a limit of detection of 1.02 nM for sodium fluorescein solution was obtained with the present LIF module, which is comparable to many of convectional and micro LIF detection systems^40–42^. Third, to ensure the compact size of the LIF module, we designed and fabricated a monolithic holder from a black ABS block to achieve the integrative assembly of all of the components in the holder including the laser diode, collimating lenses, capillary, filter, and photodiode, with a total module size of 44 × 42 × 40 mm.

We tested the detection ability of the LIF module using sodium fluorescein as model sample by alternately introducing different concentration fluorescein solutions and borate buffer (pH = 9.2) solution into the capillary. A linear relationship was obtained from 5 to 100 nM sodium fluorescein with a regression equation of F = 6.29 C + 31.8 (R^2^ = 0.999) (Fig. 2c), and a typical signal recording of 5 nM sodium fluorescein is shown in Fig. 2d. The calculated limit of detection for fluorescein is 1.02 nM. Although this detection limit is higher than those of some high-sensitive LIF systems usually with LODs from 10 pM to 10 nM^2,33,43–45^, such a detection ability could be comparable to many of reported desktop LIF systems with photodiode detectors^46,47^, or even with PMT detector^48^. Considering its extremely small size and low cost of $150, such a detection performance could be accepted for a miniaturized bioanalyzer. In the following applications including analysis of real PCR products and RFLP digestion products (see section of “*Application in analysis of KRAS gene mutation*”), the LIF module has showed sufficient performance to meet the detection requirements.

### Electronic Module for the Whole Bioanalyzer

As a palmtop instrument targeted at field analysis, the electronic module for the whole instrument should have minimized size and be capable of working standalone. So the electronic module needs a full function circuit, an integrated user interface instead of computer, and a battery power supply (Fig. 1d).

In building of the full function circuit, to simplify the circuit design and reduce the use of discrete and large-sized components as much as possible, we chose a microcontroller and an embedded system as centerpiece of circuit instead of commonly used data acquiring (DAQ) card and computer. A cost-effective 16-bit mix-signal microcontroller MSP430F1611 was selected to serve as the embedded microcontroller duo to its rich peripherals and low power property. It has an ultra-low-power architecture designed specifically for battery-powered applications, which integrates one 12-bit analog-to-digital converter (ADC12), dual 12-bit digital-to-analog converters (DAC12), and two universal serial synchronous/asynchronous communication interfaces (USART). A tiny electronic stepper motor driving chip LB1838 was chosen to replace the power-consuming stepping moto driver. The microcontroller manipulates the mini translation stage and high voltage module via the driving chip LB1838 and one built-in DAC12. The built-in ADC12 replaced the commonly used DAQ card to acquire the fluorescence signals. The signal data were processed by the controller, and the TF card was connected to the microcontroller via the built-in USART interface and served as data storage media. The entire CE analysis operation, including laser and high voltage controlling, translation stage driving, and signal acquiring, filtering, display and storage, was realized by the minimized printed circuit board with a size of 62 × 55 mm. The user interface was constructed by incorporating the TFT LCD screen and keypad, with which the electrophoresis parameters could be set and the electropherogram could be displayed on the LCD in real time.

For meeting the requirement of being powered by a battery for a portable analyzer, low power consumption components were chosen in the analyzer, including a 5 mW microcontroller, an 85 mW LCD, an 80 mW laser diode, a 200 mW stepper motor of the translation stage, and a ~70 mW mini high voltage module. As a result, a mobile phone Li-ion battery (3.7 V, 1150 mAh) could supply the analyzer a standby time for at least 10 hours with the lowest power consumption of ca. 125 mW, and a continuous working time for 4.5-hour with high-voltage of 4500 V.

### Performance of the High-Speed CE Bioanalyzer

The above modules were assembled into an integrated bioanalyzer with an entire volume of 90 × 75 × 77 mm (length × width × height), weighing ca. 300 g. The total cost of the whole instrument is ca. $500.

We optimized the present bioanalyzer and explored its multiple applications for different types of samples, including amino acids, amino acid enantiomers, proteins and DNA fragments. With the optimized conditions (i.e. spontaneous injection, 5 mM borate buffer (pH = 9.2), and 800 V/cm separation electric field strength) under the capillary zone electrophoresis (CZE) mode, a mixture of 5 μM FITC-labeled amino acids, arginine, phenylalanine and glycine, was separated within 7 s with an effective separation length of 2.6 cm (Fig. 3c). High separation efficiencies in the range of 896,000–993,000 N/m (1.01–1.12 μm plate height) were obtained, corresponding to theoretical plate numbers in the range of 23,300–25,800. Due to the short channel length and fast separation speed in the present system, no obvious Joule heating effect was observed during the separation process with high electric field strength up to 800 V/cm. We also evaluated the stability of the analyzer in continuous separation of amino acids. Good repeatabilities of retention time and peak height of the three FITC-labeled amino acids were obtained in the range of 0.22–0.26%, and 1.40–2.75% (RSD, n = 11), respectively (Fig. 3d).

**Figure 4:**
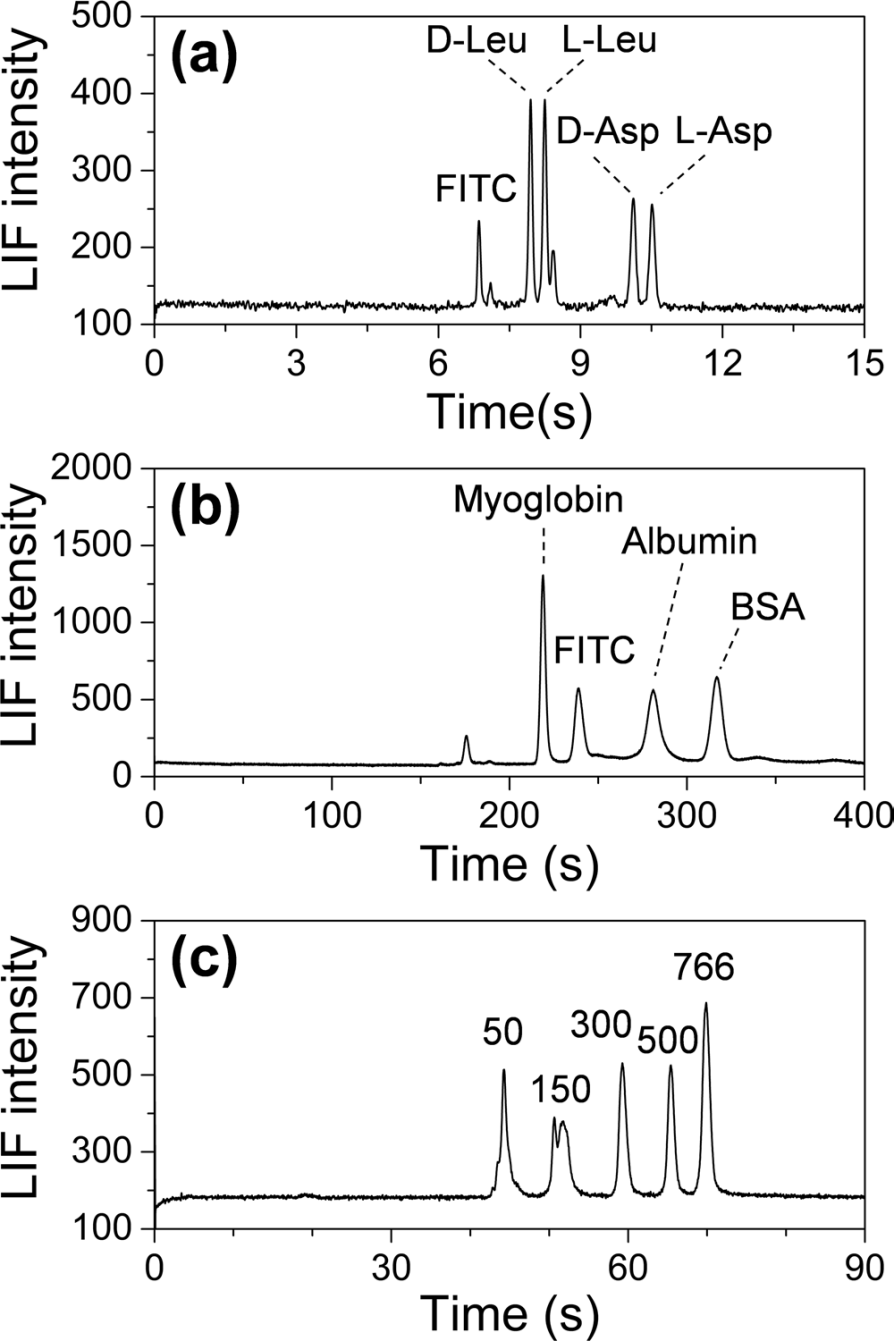
(**a**) Electropherogram of a mixture of amino acid enantiomers under the MEKC mode. Conditions: sample, mixture of 2 μM FITC-labeled D-leucine and L-leucine, D-aspartate and L- aspartate; working electrolyte, 5 mM borate buffer solution (pH 9.2) with 8 mM β-CD and 12 mM STC; translational spontaneous sample injection mode; separation electric field, 700 V/cm; (**b**) Electropherogram of three proteins under the CGE mode. Conditions: sample, mixture of 1.67 mg/mL FITC-labeled myoglonbin, albumin and BSA; translational spontaneous sample injection mode; separation electric field, 300 V/cm; (**c**) Electropherogram of a DNA ladder under the CGE mode; Conditions: sample, 1 μg/mL DNA ladder (50, 150, 300, 500, 766 bp), labeled with SYBR Green II; working electrolyte, 1 × TBE buffer, 6% PVP (m/v); electrokinetic injection mode; injection electric field, 50 V/cm; injection time, 0.5 s; separation electric field, 250 V/cm.

We also applied the bioanalyzer to analyze more challenging samples, including amino acids enantiomers under micellar electrokinetic chromatography (MEKC) mode, as well as proteins and DNA fragments under capillary gel electrophoresis (CGE) mode. With an optimized running buffer of 5 mM borate buffer (pH 9.2) containing 8 mM β-CD and 12 mM STC served as chiral selectors, two pairs of FITC-labeled amino acid enantiomers (D-leucine, L-leucine, D-aspartate and L-aspartate) were resolved in 11 s (Fig. 4a).

For separation of proteins, we adopted a commercial running buffer of SDS-MW gel buffer (Beckman Coulter, Brea, USA) to form dynamic coating in the capillary surface to suppress the protein adsorption. Myoglobin, ovalbumin and bovine serum albumin, with molecular weight of 17 kDa, 45 kDa and 66 kDa, were separated in 330 s (Fig. 4b). The separation efficiencies were in the range of 280,000–970,000 N/m, corresponding to 3.98–1.03 μm plate height. A linear relationship was obtained between the migration time and protein molecular weight (R^2^ = 0.995), which implies that the system could implement the fast determination of protein molecular weight.

### Application in Analysis of KRAS Gene Mutation

The fast separation of DNA fragments and proteins is one of the major actual application for miniaturized CE systems, especially for microchip-based CE systems^1^. To further demonstrate the potential of the present palmtop bioanalyzer in real sample analysis, we applied it in the separation of PCR products and RFLP digestion products of KRAS proto-oncogene for actual colorectal cancer (CRC) diagnosis. The analysis of KRAS gene mutation based on the PCR-RFLP method^49^ includes PCR of genomic DNA, digestion of the PCR product with restriction endonuclease Mva I, and CE separation of RFLP digestion products.

First, we optimized the analyzer in separation of a DNA ladder (50, 150, 300, 500, and 766 bp). A 6% (m/v) polyvinylpyrrolidone (PVP) solution was served as dynamic coating reagent and sieving matrix for DNA fragment separation. Due to the relatively high viscosity of PVP solution, we used electrokinetic injection method with lower field strength than separation instead of the spontaneous injection method to obtain narrow sample plug^32^. Under optimized conditions, i.e. 50 V/cm injection electric field, 0.5 s injection time, 250 V/cm separation electric field, five DNA fragments were separated in 70 s (Fig. 4c). The separation efficiencies of the DNA fragments were in the range of 730,000–1,151,000 N/m (1.37–0.87 µm plate height), corresponding to theoretical plate numbers in the range of 19,000–29,900.

**Figure 5:**
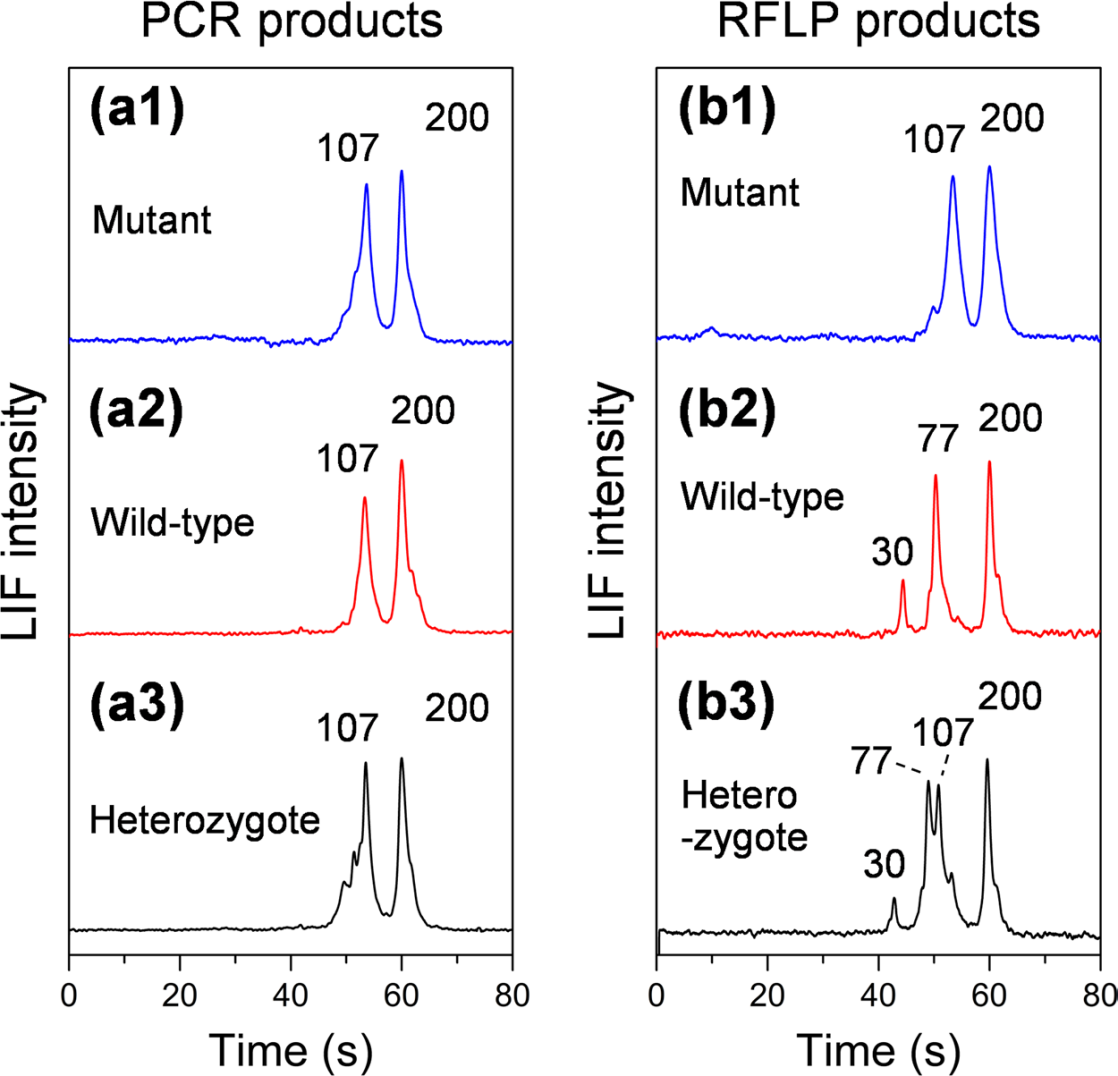
Electropherograms of PCR products (**a**) and RFLP digestion products (**b**) of KRAS proto-oncogenes in three types of CRC cells. Conditions: samples, SW480, HT29 and CCL-187 cells with KRAS gene statuses of mutant, wild-type, and heterozygote; sample DNA fragments were labeled with SYBR Green II, with 200 bp DNA fragment added as internal standard; working electrolyte, 1 × TBE buffer, 6% PVP (m/v); electrokinetic injection mode; injection electric field, 200 V/cm; injection time, 3 s; separation electric field, 200 V/cm.

We used the above-mentioned method in screening of the CRC genotype. CRC is one of the most common malignant tumors of digestive system. It has been reported that the KRAS mutation status plays a critical role in the tumorigenesis and treatment of CRC. Therefore, it has become a standard detection of gene status to CRC therapy. In this study, we used the bioanalyzer to detect the mutation of KRAS proto-oncogene. KRAS locates in the chromosome 12, and encodes a P21 protein which is closely related to tumor formation, proliferation and migration. We carried out PCR-RFLP for detecting KRAS point mutation in codon 12 of three types of CRC cells, SW480, HT29 and CCL-187 cells, with gene statuses of mutant, wild-type, and heterozygote, respectively. A 107-bp fragment of KRAS was first amplified from DNA templates extracted from the three CRC cell samples via mismatched primer PCR. Then, the PCR products were directly digested by Mva I restriction endonuclease via RFLP. Finally, the RFLP digestion products with SYBR Green I were loaded in the bioanalyzer for CE separation. To ensure the analysis reliability, we quantitatively added a 200-bp internal standard in each sample to calibrate the retention time and peak height. The electropherograms of PCR products for control and RFLP digestion products of the three cell samples are shown in Fig. 5. The resolution length (i.e. the smallest difference in DNA base pairs that can be resolved) of the present bioanalyzer was 9.6 bp for 30 bp to 107 bp DNA fragments. The PCR products of three genotype show similar results (Fig. 5a), while the RFLP digestion products have diverse results (Fig. 5b). For wild-type KRAS, the 107-bp fragment could be completely cleaved into 77-bp and 30-bp fragments by the restriction endonuclease (Fig. 5b2). However, the amplicon from the mutant KRAS template could not be digested due to the loss of recognition site and thus maintained 107 bp as the original one (Fig. 5b1). The amplicon from heterozygous template was half-digested, showing three peaks of 30 bp, 77 bp, and 107 bp (Fig. 5b3). Such results are consistent with those of routine gene mutation analysis method with slab gel electrophoresis, which demonstrates the present bioanalyzer could be used to distinguish whether a KRAS gene mutates and which mutation status it has on the basis of the fast CE separation results. With the present instrument, separation time was evidently reduced from tens of minutes by slab gel electrophoresis to 1.5 min, which substantially speeds up the identification of the PCR and digestion products. Furthermore, the compact size of the palmtop instrument also allows it to be conveniently used as a handy tool, such as a pipette, to rapidly get the PCR identification results on-site once after the PCR amplification experiment.

## Conclusion

In the present work, we developed an integrated palmtop high-speed CE bioanalyzer capable of performing high-speed and high-efficiency CE analysis with a total dimension of 90 × 75 × 77 mm, which reached higher level in system miniaturization and integration than the previously-reported systems^38–46^. Actually, to our knowledge, it is the smallest high-speed CE apparatus with LIF detection reported in the literatures. It is commendable that the analyzer was built mostly using simple and cheap components, and thus had extremely low instrument cost of $500. The effectiveness and broad application potential of the palmtop bioanalyzer have been extensively demonstrated in analysis of multiple different types of samples including actual analysis of gene mutation, with separation performances comparable to other miniaturized and large-size high-speed CE instruments.

More importantly, in the development of the palmtop bioanalyzer, we used a strategy of minimalist miniaturization differing from the currently-used ones in most reported miniaturized CE systems. This strategy combines minimalistic system design and low-cost system construction, to simplify the system structure as much as possible, and thus to reduce the difficulty and cost in instrument miniaturization and application, with comparable analytical performance to desktop systems. It mainly relies on the essential understanding to the working principle of the analytical system and the use of previous innovation achievements (e. g. the translational spontaneous injection^29,30,36^ and 45° deviated orthogonal LIF detection^37^) in system design, as well as the employment of various commercially-available small components currently-used in multidisciplinary fields (e. g. the mini-stage and collimating lens) and low-cost fabrication methods in system construction. The experimental results successfully demonstrated the feasibility and effectiveness of this strategy in miniaturized instrument development. Such a strategy provides a novel train of thought and solution for development of miniaturized analytical instruments. We expect it could be adopted in the miniaturization of other types of analytical instruments, and may also has potentials in promoting the widespread popularization of instrument miniaturization study and products in various routine laboratories due to its simple and low-cost features.

For a miniaturized instrument, usually its analytical performance will decrease to some extent compared with routine benchtop instruments, due to the compromise between system miniaturization and performance. In the present work, since the main objective is to achieve the miniaturization of high speed CE instrument, we did not carry out further detailed optimization to the separation conditions. Also due to the uses of some miniaturized and low-cost modules, the analytical performance of the instrument could not reach the highest levels of separation speed and efficiency as the desktop CE systems. Even though, its separation performance is still comparable to many of microchip-based high-speed CE systems, and is capable of meeting the demand of the performed application experiment.

For the further application of the present palmtop bioanalyzer, since we have applied it in electrophoretic separations of multiple types of biochemical samples with good flexibility for different CE modes of CZE, MEKC and CGE, it could be used as a quick and easy-to-use handy tool in routine biochemical laboratories for fast separation of protein and DNA fragment samples, which may have potential to partially substitute the widely-used traditional slab gel electrophoresis devices. In addition to the application in laboratories, such a miniaturized instrument also has broad application prospects in field analysis and *in-situ* analysis, such as point of care testing (POCT) or companion diagnostics for disease diagnosis, on-site environmental analysis, as well as aeronautic and astronautic analysis, due to its properties of portable size, low cost, long-term independently working and high-resolution separation.

## Methods

### Chemicals and materials

All reagents were of analytical grade, unless mentioned otherwise. Amino acids were purchased from Kangda Amino Acid Works (Shanghai, China). Fluorescein isothiocyanate (FITC), polyvinylpyrrolidone and all proteins were obtained from Sigma-Aldrich (St. Louis, USA). 5 × TBE buffer (0.225 M Tris/boric acid, 0.05 M EDTA) for DNA separation was purchased from Sangon Biotech (Shanghai, China). SYBR Green I used as a double-stranded DNA binding fluorescent dye was purchased from Xiamen Zeesan Biotech (Xiamen, China). FastDigest Mva I for DNA digestion was purchased from Thermo Fisher Scientific (Waltham, USA). A 200 bp DNA fragment as an internal standard in DNA separation was obtained from Beijing Biomed (Beijing, China), and other reagents for DNA separation were purchased from Sinopharm Chemical Reagent (Shanghai, China). All of aqueous solutions were prepared within deionized water, then filtered through 0.22 μm Millipore filters (Darmstadt, Germany), and stored at 4 °C.

### Setup of the bioanalyzer

The palmtop bioanalyzer (Fig. 1) consists of multiple modules including automated sample injection, CE separation, orthogonal LIF detection, instrument control and display, data acquisition, processing and storage, and battery power supply.

A short fused-silica capillary (3.8 cm length, 360 μm o.d., 50 μm i.d.; Reafine Chromatography Co., Yongnian, China) with a tapered tip^37^ (50 μm tip size, fabricated as previously reported^37^) was used as separation channel. Slotted vials produced by cutting a 1–2 mm slot on the bottom of each 200 μL PCR tube (Axygen, Union City, USA) were used for loading sample, running buffer and waste solutions. For achieving automated sample injection, the sample and running buffer vials were horizontally fixed on a mini translation stage (F6N09, Sanyo, Osaka, Japan), which is originally used for autofocus in a digital camera. A miniature high-voltage module (Q60N, EMCO Co., Sutter Creek, USA) was adopted to provide variable voltage in the range of 0 to −6000 V for sample injection and CE separation, through two Pt electrodes inserted into the running buffer/sample and waste vials, respectively.

In the LIF detection module, a 450 nm laser diode (PL 450B, 80 mW, Osram, Munich, Germany) was used as light source, and a photodiode (S8745-01, Hamamatsu photonics, Hamamatsu, Japan) was used as an optical detector. The laser beam was focused on the channel center of the capillary by a collimating lens (0.30 NA, Leimei Electronics, Guangzhou, China) which was originally used for laser collimation for commercial laser diodes, with a focusing spot size of ca. 10 μm. The excited fluorescence collected by another collimating lens and filtered by a 510 nm band-pass filter (BF 510, HB Optical technology Co., Shenyang, China), was detected by the photodiode placed at the plane perpendicular to the laser beam with a detection angle of 45° to the capillary. The capillary and all of the optic components in the LIF module were installed in an acrylonitrile butadiene styrene (ABS) holder (Fig. 2a,b) fabricated by a computer numerical control machine (AM-01, AMO Electronics., Dongguan, China). In the holder, the components were placed in their corresponding positions and collimated automatically without further optical alignment.

The block diagram of the electronic module of the instrument is shown in Fig. 1d. A microcontroller (MSP430F1611, Texas Instruments, Dallas, USA) was served as the centerpiece to control the whole CE operation automatically. A built-in 12-bit analog-to-digital converter (ADC12) was utilized to acquire the fluorescence signal detected by the photodiode. A built-in 12-bit digital-to-analog converter (DAC12) was used to control the mini high voltage module (Q60N, EMCO, Sutter Creek, USA). An amplifier (AD623, Analog Devices, Boston, USA) was used to further amplify the output of the photodiode. A TransFlash (TF) card (16 GB, Sandisk, Milpitas, USA) was connected to microcontroller via a serial peripheral interface (SPI) for data storage. The laser diode was supplied with a constant current source which was controlled by the microcontroller directly. The stepper motorized translation stage was controlled by the microcontroller via a driver chip (LB1838, Sanyo, Osaka, Japan). A 320 × 240 pixels thin film transistor liquid crystal display (TFT-LCD) screen (Yaoyu Co., Shenzhen, China) and a four-button keypad were incorporated to construct the user interface. An USB interface for communication also was provided by a conversion chip (FT232, Future Tech., Glasgow, United Kingdom) which was connected to the universal asynchronous receiver/transmitter (UART) port of the controller. The circuit was powered by a 3.7 V Li-ion battery (BL-4 U, Pisen, Shenzhen, China). The photodiode and high voltage module were supplied by ±12 V voltages which were converted by a power convert module (E0312T, Mounsun, Guangzhou, China), and the other part of the circuit was supplied by 3.3 V which was regulated by a low dropout regulator (SP6203, Sipex, Milpitas, USA).

Finally, all of the modules were installed into a small instrument case with a size of 90 × 75 × 77 mm (length × width × height). The LCD screen and control buttons were installed on the top panel of the case cover.

### Procedures

Before experiments, the capillary was inserted into sleeve tubes and fixed on the holder. Through connecting to injectors, it was rinsed in sequence by 1 M NaOH, water and running buffer solution, each for 10 min. A 50 μL sample solution and a 100 μL running buffer were loaded into the corresponding slotted vials by a pipette, respectively. Then the instrument cover was closed, and the instrument was switched on. Multiple parameters such as sample injection mode, separation voltage, and running time, were set via the control buttons and the LCD screen. The real-time light intensity signal detected by the LIF module was displayed in real-time on the LCD screen and the data was stored in the TF card in the analyzer. For sample injection, the mini translation stage carrying the sample and running vials linearly moved under control of the set program, to allow the capillary inlet tip first to enter into the sample vial through its slot, with a voltage applied between the capillary inlet and outlet under the electrokinetic injection mode or without voltage applied under the spontaneous injection mode, and then shift back to the running buffer vial again. When the capillary inlet immersed into the running buffer in the vial, a separation voltage was added between the capillary inlet and outlet to start CE separation. A movie clip (Movie S1) showing the working process of the bioanalyzer is provided in Supporting Information.

### Safty considerations

When building and operating the LIF detection system, laser protective goggles should be worn to avoid eye damage.

## Electronic supplementary material

Supplementary Information
